# The Search for the Soul

**Published:** 1921-06-04

**Authors:** 


### THE SEARCH FOR THE SOUL.

There is probably no more interesting chapter in the history of thought than that which deals with the speculations as to soul and mind. We are only too apt to depreciate the strivings after knowledge of our predecessors, forgetting that their opportunities of acquiring accurate information were limited by the conditions in which they lived. Research into the ultimate constitution of the nervous system, for example, was not possible until after the discovery of the microscope; nor could the demonstration of the existence of micro-organisms be carried out prior to that period. The result was that hypothesis and speculation held the field, and so attached did thinkers become to these products of their cogitation that they resisted efforts made to enlighten them by those who had verified knowledge by observation and by experiment. Their resistance was by no means always of the passive type; only too often they actively resented the attempt to disabuse their minds of error, and, as they were usually in the majority, they were able to make things very unpleasant for the innovators! However, we must not be hypercritical of their actions in this respect, for it seemed to them, in many instances, that the supplanting of their ideas was subversive of discipline, and therefore dangerous to the State. It appears to be that each generation must work out its own salvation. Yet historical review of the matter is invaluable in helping us to avoid some of the errors of the past, though most of us are too busy with the present to spare time for retrospect, or, at least, we think we are. It is true that to study systems of philosophy and to wade through the morasses of verbiage such an investigation would entail is an almost impossible task, even to those who have the inclination to do so.

We may, however, economise our time by consulting such books as G. H. Lewes' "History of Philosophy" or his "Biographical History of Philosophy," to mention no others. But there is a book recently published which epitomises admirably the speculations as to mind and soul—Dr. Hollander's "In Search of the Soul." In addition to a conspectus of the theories from primitive times onwards, he gives an extr[illegible]nely interesting account of the practical bearings they have had, as, for example, in the treatment of the insane, the witchcraft delusion, and of the general influence on the progress of research, especially in regard to the nervous system. It is heartening to see how, in spite of many halts and occasional retrogressions, the march of progress towards rational belief has continued. Airy abstractions have been gradually superseded by more solid arrays of physical facts and the ground prepared for the synthesis of a real psychology and, following in its train, a science of ethology—the study of human conduct in all its aspects.

We see more and more clearly that it is no mere hobby for web-spinning individuals who endeavour to excogitate, chiefly by introspection, a system of thought which is satisfactory on paper. It is the practical concern of us all, and upon it depends whether or not we shall be able to formulate proper rules for the maintenance of health, for the correct treatment of mental disorders and of crime, and for, indeed, our conduct in general. A right understanding of the important reciprocal factors—temperament and environment, nature and nurture—is the only thing that can lead to social betterment. Copy-book maxims and pious platitudes have done little to help mankind, and ideal schemes of morality remain divorced from workaday practice. They have failed because they were formulated by people who were greatly ignorant of the essential factors which are the springs of human conduct, the mechanism behind the mind.

